# Children with respiratory tract infections in Swedish primary care; prevalence of antibiotic resistance in common respiratory tract pathogens and relation to antibiotic consumption

**DOI:** 10.1186/s12879-017-2703-3

**Published:** 2017-09-04

**Authors:** Mia Tyrstrup, Eva Melander, Katarina Hedin, Anders Beckman, Sigvard Mölstad

**Affiliations:** 1 Department of Clinical Sciences, Family Medicine, Lund University, Malmö, Sweden; 2 Department of Translational Medicine, Lund University, Malmö, Sweden; 3 Regional Centre for Communicable Disease Control, Malmö, Skåne County Sweden; 4 Futurum- Academy for Health and Care, Region Jönköping County, Sweden

## Abstract

**Background:**

The majority of antibiotics consumed in developed countries are prescribed in primary care. However, little is known about resistance levels in the primary care population.

**Method:**

Nasopharyngeal cultures were obtained from children, 0-10 years of age, seeking care at their Primary Health Care Centre with symptoms of respiratory tract infection. Parental questionnaires were used to retrieve information about the child’s previous antibiotic consumption.

**Result:**

Cultures from 340 children were gathered. The level of resistant *Haemophilus influenzae* was low and the prevalence of penicillin non-susceptible pneumococci (PNSP MIC ≥ 0.125 mg/L) was 6% compared to 10% (*p* = 0.31) in corresponding cultures from children diagnosed at the local clinical microbiology laboratory. Antibiotic treatment within the previous 4 weeks predisposed for resistant bacteria in the nasopharynx, OR: 3.08, CI 95% (1.13-8.42).

**Conclusion:**

Low prevalence of PNSP supports the use of phenoxymethylpenicillin as empirical treatment for childhood upper respiratory tract infections attending primary care in our setting. It is important that studies on resistance are performed in primary care populations to evaluate data from microbiological laboratories. Recent antibiotic treatment increases risk of bacterial resistance in children and continuous work to reduce unnecessary antibiotic prescribing should be prioritised.

**Electronic supplementary material:**

The online version of this article (10.1186/s12879-017-2703-3) contains supplementary material, which is available to authorized users.

## Background

In Swedish Primary Health Care (PHC) about one-third of all consultations are regarding infections, of which some 60% concern respiratory tract infections (RTIs), such as acute otitis media (AOM), sinusitis and pneumonia [1]. A common pathogen causing these conditions in children is *Streptococcus pneumoniae* [2, 3]. *S. pneumoniae* can also cause severe invasive disease, especially in young children and the elderly. In Sweden about 2-3 children under the age of five die due to invasive pneumococcal disease each year. Since the pneumococcal vaccine was introduced in Sweden in 2009 the incidence of serious pneumococcal infections in children under the age of two has been reduced by 70% [4]. In healthy children attending daycare in Sweden, the prevalence of *S. pneumoniae* in the nasopharynx (NP) is about 60% [5] and the prevalence decreases with age from the second year of life [6, 7] to about 5% in an adult population [8]. Phenoxymethylpenicillin (Penicillin V) is the drug of choice for empirical treatment of respiratory tract infections in Scandinavia and is recommended in the national guidelines. In therapeutic failure or if an appropriate culture shows growth of penicillin non-susceptible pneumococci (PNSP, MIC ≥ 0.125 mg/L) or *Haemophilus influenzae* second-line treatment with amoxicillin is advised.

The vaccination program in Sweden includes *Haemophilus influenzae* type B- and the 13- valent pneumococcal vaccine. The current coverage for at least three doses of these vaccinations in Sweden is 97%, calculated on all children registered with a Primary Health Care Centre (PHCC), which include 99% of all 2-year old children registered in Sweden in 2016 [4].

In 1967 the first report of a strain of penicillin non-susceptible *S. pneumoniae* (PNSP MIC ≥ 0.125 mg/L), was encountered in Australia [9] and in the following decade reports of PNSP followed from many other countries. High frequencies of PNSP, around 20% in invasive isolates were noted in central and southern parts of Europe, such as in France, Spain and Hungary in 2011 and in Germany, the Netherlands and the Nordic countries frequencies below 5% have been reported [10]. Iceland had an increase of PNSP at the beginning of the 1990s in hospital isolates to a level of 20% in 1993 [11], while the level in PHC was about 10% during the same year [12]. The frequency of PNSP has been found to correlate to antibiotic prescription at an individual as well as at community level [11–14].

In the early 1990s, an increase of PNSP (MIC ≥ 0.125 mg/L) from 3 to 11% was also noted in Sweden [15]. An epidemic spread of several clones of PNSP was seen in the southernmost part of Sweden and the South Swedish Pneumococcal Intervention Project was launched to curb the spread [16]. Since then, a low prevalence of PNSP (4%) has been noted in surveys of healthy toddlers in Sweden [17], but there has been an increase in incidence from 3.7% in 1994 to 9.8% in 2015 in the data reported from the microbiological laboratories (ResNet) [18].

*Haemophilus influenzae* is the second potential pathogen targeted when treating RTIs with antibiotics, causing epiglottitis and invasive disease including pneumonia. However, since the vaccination against *H.influenzae* type b (Hib) was introduced into developed countries these severe conditions have decreased substantially, though they are still present in developing countries where vaccination coverage is low [19]. *H.influenzae* colonises the NP of up to 50% of healthy children [20] and are responsible for about one-third of all episodes of AOM in children [21].

The first report of beta-lactamase producing *H.influenzae* came in 1974 [22]. Penicillin resistance in *H.influenzae* is most often conferred by production of beta-lactamase, or by chromosomally mediated mechanisms, called beta-lactamase negative ampicillin resistant *H.influenzae* (BLNAR). Some *H.influenzae* strains have both of these resistance mechanisms. Since 2013, beta-lactamase producing *H.influenzae* has shown a decreasing trend, with a prevalence of around 16% in 2015 and BLNAR, with an increasing trend and a prevalence of 19% in 2015 in Sweden [18]. The trends are similar in other countries [23]. Around the world, the prevalence of beta-lactamase producing *H.influenzae* varied between 5 and 39% in 2003 [24] and the prevalence of BLNAR around 10% in Europe in 2000, but up to 40% in Japan [23] in data from microbiological laboratories.

National data on antibiotic resistance in Sweden are based on cultures from NP swabs from both hospital and primary care patients. The 25 microbiological laboratories each annually report ≥100 consecutive strains per pathogen to the national register Res-Net (Swedres/ SWARM 2016, p.110-111, www.folkhalsomyndigheten.se). In PHC it is only recommended to perform swabs on patients with AOM when first-line treatment has failed or in recurrent infections, and not in children seeking primary care with their initial symptoms of RTI. The true level of bacterial resistance in children with RTI symptoms consulting PHC remains unknown and the results from the laboratory may therefore not be valid in a PHC setting.

The aim of this study was to evaluate the prevalence of PNSP and beta-lactamase producing *H.influenzae* and BLNAR in children with symptoms of RTI presenting at Primary Health Care Centres (PHCCs) and to compare our findings with those of routine microbiological lab data.

Furthermore, we wanted to investigate the relation between presence of resistant bacteria (PNSP, beta-lactamase producing *H.influenzae*, BLNAR) and previous antibiotic consumption and other risk factors.

## Methods

We performed a cross-sectional study on children, 0-10 years of age, registered at 12 different PHCCs in the county of Scania, in the southernmost part of Sweden. The sizes of the PHCCs varied from two up to 15 serving physicians. The PHCCs were chosen to represent both urban (four PHCCs) and rural (eight PHCCs) areas of the region, including the city of Malmo with approximately 320.000 inhabitants. The antibiotic prescription rates of the PHCCs were similar to the average prescription rate of the county. In 2013 the county of Scania had a population of 1.274.069 inhabitants, of which 167.484 were children aged 0-10 years [25]. Aiming to collect 100 pneumococcal isolates, and based on 2013-2014 regional microbiological laboratory data with the carriage rate of 30% in 0-10 year olds (E. Melander, personal communication, May 30, 2016) we estimated a need for approximately 400 NP specimens.

### Data collection

Children, aged 0-10 years, consulting their PHCC with symptoms of RTI, between 1st Nov 2013 until 30th April 2014 and 1st Nov 2014 until 30th April 2015 were included. Information about the study and a written invitation was given to the parents at arrival at the clinic and informed parental consent was obtained for each case. Lack of knowledge of the Swedish language was the only exclusion criterion.

### Questionnaires

The parents were asked to fill out a questionnaire regarding factors believed to affect bacterial resistance, such as the child’s age, antibiotic consumption over the last year and specifically during the last 4 weeks, type of day care, number of siblings, travel abroad, hospital admissions etc. (Additional files 1 and 2). The same patient was only included once. The individual antibiotic consumption was evaluated via the questionnaire filled out by the parents.

### Sample collection and bacterial analysis

NP specimens were obtained in a standardised manner by trained laboratory personnel at the PHCCs. A swab (ESwab ™ Liquid Amies Collection and Transport System, COPAN) [26] was taken from the rear wall of the nasopharynx, where the stick was kept still for 10 s before being withdrawn. The test swab was kept in a refrigerator until being sent to the Department of Clinical Microbiology in Malmö/Lund where it was analysed for *S.pneumoniae*, *H.influenzae*, *Moraxella catarrhalis* and beta-haemolytic streptococci group A, according to national recommendations [27]. Antibiotic susceptibility testing was performed according to recommendations of the EUCAST [28]. Isolated *S.pneumoniae* were screened for penicillin resistance by disc diffusion test using 1-microgram oxacillin discs. Minimum inhibitory concentration (MIC) for benzylpenicillin was determined by E-test in isolates with inhibition zones <20 mm. *S.pneumoniae* with MIC of ≥0.125 mg/L was considered penicillin non-susceptible (PNSP). Screening for beta-lactam resistance in *H.influenzae* was performed by disk diffusion test using benzylpenicillin discs. If found to be benzylpenicillin resistant, a beta-lactamase test was performed (Nitrocefin). For detection of chromosomal penicillin resistance (BLNAR), a disk diffusion test was used with a beta-lactamase stable cephalosporin (cefaclor) as an indicator.

In this paper, colonization with resistant bacteria was defined as the presence of PNSP and/or *H.influenzae* strains with beta-lactamase production and/or *H.influenzae* with chromosomal resistance, (BLNAR).

### Statistical analysis

The main outcome was the presence of PNSP and resistant *H.influenzae* in the NP culture. Questionnaire and laboratory data were collected in Microsoft Excel and statistical analysis was performed using SPSS 22.0 software (IBM, Armonk, NY, USA). Descriptive statistics are presented as numbers and proportions. Comparisons between proportions for categorical variables in two independent groups were performed using the two-sided χ^2^-test. Multiple logistic regressions were used to model the relationship between the outcome variable (carriage of resistant bacteria) and several independent variables (risk factors for carriage of resistant bacteria retrieved from the parental questionnaire).

## Results

### Baseline data

**Fig. 1:**
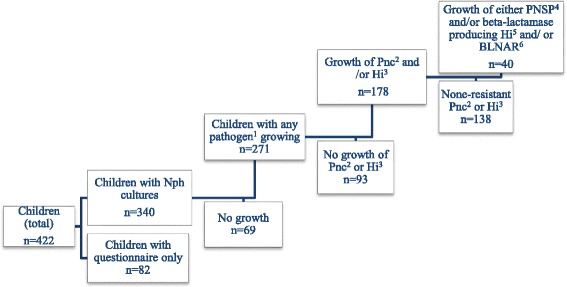
Flow chart of children in the study. ^1^ Either *Streptococcus pneumoniae, Haemophilus influenzae, Moraxella catarrhalis* or Group A Streptococci. ^2^ *Streptococcus pneumoniae*. ^3^ *Haemophilus influenzae*. ^4^ penicillin non-susceptible *Streptococcus pneumoniae*. ^5^ beta-lactamase producing *Haemophilus influenzae*. ^6^ beta-lactamase negative ampicillin resistant *Haemophilus influenzae*

Of the 12 PHCCs, eight provided 90% of the NP cultures. We aimed at collecting 400 cultures but managed 340 due to declining recruitment. Altogether, 422 children were recruited, of which 340 participated with NP cultures and the parental questionnaire. A total of 82 children did not accept having nasopharyngeal culture taken (Fig. 1). The baseline characteristics of the children with and without a nasopharyngeal culture taken were similar (Table 1). The median age in both groups was 2.0 years (IQR = 1-5).

**Table 1:** Demographic and clinical data of study population

	Number (%)			
Variable	All children with Nph^a^ culture (*n* = 340)	Children with growth of either Pnc^b^ and/ or Hi^c^ (*n* = 178)	Children with growth of either PNSP^d^ and/ or beta-lactamase producing Hi^e^ and/ or BLNAR^f^ (*n* = 40)	Questionnaire only (*n* = 82)
Female	162 (48)	92 (52)	14 (35)	41 (50)
Age (0-5 years)	275 (81)	160 (90)	38 (95)	65 (79)
Hospital care last 6 months	11 (3)	7 (4)	2 (5)	1 (1)
Abroad last 3 months	81 (24)	43 (24)	10 (25)	21 (26)
Parents smoking	43 (13)	16 (9)	2 (5)	10 (12)
Day care:				
Attending day care centre	224 (67)	137 (77)	33 (83)	56 (68)
Attending school	60 (18)	7 (4)	1 (3)	16 (20)
Home	52 (15)	24 (14)	6 (15)	9 (11)
Respiratory tract disease (asthma/ allergy)	34 (10)	20 (11)	1 (3)	9 (11)
Pneumococcal vaccination:				
Don’t know	94 (28)	40 (22)	9 (23)	30 (37)
Yes	218 (64)	128 (72)	28 (70)	46 (56)
No	28 (8)	10 (6)	3 (8)	6 (7)
Number of antibiotic treatments during last 12 months:				
> 3	22 (6)	11 (6)	4 (10)	6 (7)
1-2	98 (29)	46 (26)	12 (30)	19 (23)
None	182 (54)	105 (59)	21 (53)	57 (70)
Antibiotic treatment within the last 4 weeks	32 (9)	15 (8)	7 (18)	5 (6)

Missing data were 20% regarding “Number of antibiotic treatments during last 12 months”, similar in all groups. In the other variables missing data were <10% in all groups

^a^Nasopharyngeal culture

^b^ *Streptococcus pneumoniae*

^c^ *Haemophilus influenzae*

^d^penicillin non-susceptible *Streptocuccus pneumoniae*

^e^beta-lactamase producing *Haemophilus influenzae*

^f^beta-lactamase negative ampicillin resistant *Haemophilus influenzae*

Of the 340 children, 86 (25%) carried *S.pneumoniae*, 129 (38%) *H.influenzae*, 185 (54%) *M.catarrhalis*, 24 (7%) beta haemolytic streptococci group A and 69 (20%) were culture negative or carried non-target organisms (Table 2). Of the 86 isolates with *S.pneumoniae,* five (6%) were PNSP all with MIC = 0.125 mg/L. Three of these PNSP isolates also showed resistance to trimethoprim, one showed additional resistance to tetracycline and one additional resistance to tetracycline, erythromycin and clindamycin. Resistance data are presented in Table 2.

**Table 2:** Data on isolated bacteria in nasopharyngeal cultures of children 0-10 years during November 2013-April 2014 and November 2014-April 2015, in the study and in routine laboratory data

	Our data (primary care)	Routine laboratory data (primary care and hospital care)
Number of cultures	340	1854
*Streptococcus pneumoniae*	86 (25%)	468 (25%)
PNSP^a^ (MIC ≥ 0.125 mg/L)	5 (6%) All with MIC = 0.125 mg/L	47 (10%) Of which: 32 MIC = 0.125 mg/L 6 MIC = 0.25 mg/L 4 MIC = 0.5 mg/L 4 MIC = 1 mg/l 1 MIC = 2 mg/L
Erythromycin resistant	4 (5%)	32 (7%)
Clindamycin resistant	3 (3%)	25 (5%)
Tetracycline resistant	4 (5%)	23 (5%)
Trimethoprim resistant	7 (8%)	53 (11%)
*Haemophilus influenzae*	129 (38%)	747 (40%)
Beta-lactamase producing	21 (16%)	96 (13%)
BLNAR^b^	16 (12%)	57 (8%)
*Moraxella catarrhalis*	185 (54%)	1524 (82%)
*Group A streptococci*	24 (7%)	138 (7%)

^a^penicillin non-susceptible pneumococci

^b^Beta-lactamase negative ampicillin resistant *Haemophilus influenzae*

### Factors associated with bacterial resistance

When analysing the 340 children from which NP cultures were taken, we found that having received antibiotic treatment (predominately Penicillin V) within the previous 4 weeks predisposed for resistant bacteria (OR = 3.08, 95% CI (1.13-8.42). Antibiotic consumption during the last year did not significantly affect the prevalence of resistant bacteria in our analysis. But, since it could interact with the variable antibiotic consumption the previous 4 weeks we decided to exclude it in the regression analysis. The risk of carrying resistant bacteria also decreased with age (OR = 0.75, 95% CI (0.57-0.99) (Table 3).

**Table 3:** Risk factors for carriage of resistant bacteria* among children aged 0-10 years with respiratory tract symptoms in Primary Health Care. (*n* = 340)

Variable	Crude OR (95% CI)	Adjusted **OR (95% CI)
Male sex	1.81 (0.91-3.60)	
Age (per year)	0.81 (0.69-0.95)	0.75 (0.57-0.99)
Hospital care last 6 m	1.70 (0.35-8.14)	
Abroad last 3 m	1.08 (0.50-2.31)	
Parents smoking	0.33 (0.08-1.43)	
Attending day care centre	2.69 (1.15-6.29)	2.63 (0.91-7.66)
Respiratory tract disease (asthma/ allergy)	0.20 (0.03-1.47)	
Pneumococcal vaccination (yes)	1.23 (0.35-4.34)	
Antibiotics previous 4 weeks	2.33 (0.94-5.81)	3.08 (1.13-8.42)

Missing data regarding the variables were <10% in the children with, as well as, in the children without resistant bacteria

*Growth of either penicillin non-susceptible pneumococci (PNSP) and/ or beta-lactamase producing *Haemophilus influenzae* and/or beta-lactamase negative ampicillin resistant *Haemophilus influenzae* (BLNAR)

**Method: Backward Stepwise (Wald) Step 7

In 178 (52%) of the cultures, there was growth of either *S.pneumoniae* or *H.influenzae* or both, of which 40 (22%) isolates showed some sort of resistance to beta-lactam antibiotics, either PNSP*,* and/or beta-lactamase producing *H.influenzae* and/or BLNAR (Fig. 1).

When analysing only the 178 children with growth of either *S. pneumoniae* or *H. influenzae,* or both, we noted that boys were more commonly colonised with resistant bacteria (OR = 2.95, 95% CI (1.25-6.98) (Table 4). No significant association was found between resistance and hospital care, travelling abroad, parental smoking, day care attendance, respiratory tract disease (asthma/allergy) or pneumococcal vaccination (Tables 3 and 4).

**Table 4:** Risk factors for carriage of resistant bacteria among children aged 0-10 years with respiratory tract symptoms in Primary Health Care and growth of either *Streptococcus pneumoniae* and/or *Haemophilus influenzae.* (*n* = 178)

Variable	Crude OR (95% CI)	Adjusted *OR (95% CI)
Male sex	2.41 (1.16-5.02)	2.95 (1.25-6.98)
Age (per year)	0.85 (0.70-1.04)	0.77 (0.58-1.02)
Hospital care last 6 m	1.40 (0.26-7.50)	
Abroad last 3 m	1.06 (0.47-2.40)	
Parents smoking	0.47 (0.10-2.14)	
Attending day care centre	1.54 (0.63-3.80)	
Respiratory tract disease (asthma/ allergy)	0.17 (0.02-1.28)	0.17 (0.02-1.38)
Pneumococcal vaccination (yes)	0.65 (0.16-2.69)	
Antibiotics previous 4 weeks	3.45 (1.17-10.19)	

Missing data regarding the variables were <10% in the children with, as well as, in the children without resistant bacteria

*Method: Backward Stepwise (Wald) Step 7

### Comparison with data from the local clinical microbiology laboratory

The prevalence of *S.pneumoniae* and *H.influenzae* were similar to the data from the local clinical microbiology laboratory for the same period and age group (Table 2). We could not identify any statistically significant difference in the prevalence of PNSP (*p* = 0.31), beta-lactamase producing *H.influenzae* (*p* = 0.33) or BLNAR (*p* = 0.08) between our community study and the data from the local clinical microbiology laboratory (Table 2).

## Discussion

### Main findings

We found a low prevalence of PNSP and resistant *H.influenzae* among respiratory tract pathogens in children with symptoms of RTIs in primary care, indicating that phenoxymethylpenicillin is valid as empirical treatment for these patients, if antibiotics are required. We noted no significant differences in prevalence of PNSP and resistant *H. influenzae* in our results compared to the local microbiological laboratory. Studies based on primary care populations are important for validation of resistance measures from microbiological laboratory findings due to differences in case-mix. Treatment with antibiotics, within the previous 4 weeks predisposed for resistant bacteria supporting continued efforts to rationalise antibiotic use.

### Strengths and weaknesses

Previous attempts to investigate bacterial resistance in RTIs in primary care have studied populations containing a mix of children attending PHCCs as well as those seeking care at hospital emergency rooms [29, 30]. The strength of this study is that the population consists of primary care patients only. Bearing in mind that most antibiotics are prescribed in primary care, monitoring resistance levels in this population should be of importance to appreciate the true burden of resistance as well as for the development of guidelines and recommendation of empirical antibiotic choice. Based on the carriage rate of *S.pneumoniae* in Swedish children we estimated a need of 400 cultures to identify about 100 isolates of *S.pneumoniae*, but we settled with 340 cultures and 86 isolates due to a declining recruitment rate. We chose to recruit during the winter season because the consultations for RTIs are more common at that time of the year. We do not believe that the few cases missed during the rest of the year would affect our result. We deem the risk of patient selection bias to be low because although we received a variable number of cultures from the different PHCCs, indicating difficulties in recruiting patients, we noted that most parents were willing to participate in the study once they were asked. Furthermore, the only exclusion criterion was lack of knowledge of the Swedish language. Ongoing treatment with antibiotics was not asked about, and therefore not an exclusion criterion. We had a good response rate regarding antibiotic use the previous 4 weeks, which to most parents include whether the child are on any current antibiotic treatment. The use of routine sample collection and well-established methods for analysis of NP cultures should ensure the reliability of the results.

The number of antibiotic prescriptions during the last year was difficult for some parents to answer for in the questionnaire and therefore we saw around 20% missing data regarding this question. However, since the missing data were equally distributed in the groups, with and without resistance, it should not affect the regression analysis.

The association between antibiotic consumption during the previous 4 weeks and bacterial resistance showed an OR with a wide 95% confidence interval due to a small number of children that had taken antibiotics within the 4 weeks prior to the culture and a small number with beta-lactam resistant strains. Although there is some insecurity in the result due to the small sample size it should be possible to infer to similar populations.

To differentiate whether our results represent colonisation or infection is not possible. The fact that the children all presented with symptoms of RTIs, such as fever, cough, sore throat, earache or runny nose supports the theory of it representing an infection, but, the culture might not represent the etiological agent. We did not perform any respiratory virus testing, which could explain some of the symptoms. Treatment information and outcome data could also have helped in the differentiation between colonisation and infection, although most RTIs, whether caused by bacteria or virus are self-healing. Since serotyping of the pneumococci was not performed we don’t know if they were covered in the 13-valent vaccine used in Sweden.

### Previous studies

We found that 25% of the children seeking care at their PHCC for RTI symptoms and who were recruited to our study carried *S.pneumoniae* and 38% carried *H.influenzae.* These figures were in line with other recent European and American studies [7, 29, 31, 32]*.*

By contrast, the level of PNSP (MIC ≥ 0.125 mg/L) was lower in our study than in many other countries [24, 32, 33]. PNSP levels are known to be correlated to antibiotic consumption [6]. Over the last 20 years, great reductions in antibiotic prescribing for RTIs in Swedish PHC have been achieved [34], which is likely to have played an important role in the reduction of PNSP over time. Swedish guidelines for RTIs recommend phenoxymethylpenicillin as first line treatment to target the pneumococci, and the low level of resistance found in this study supports that recommendation. By contrast, the prevalence of beta-lactamase producing *H.influenzae* does not seem equally influenced by the reduced levels of antibiotics. The beta-lactamase producing *H.influenzae* has remained between 15 and 18% in Sweden since 2007 and we see a continuous increase in BLNAR [18]. Previously, we have seen a similar development for *Moraxella catarrhalis,* of which now 95-98% is beta-lactamase producing in every setting [24, 33].

Antibiotics have been shown to affect our bacterial flora so that penicillin sensitive pneumococci decreases 1 month after antibiotic therapy [7] as does penicillin sensitive *H.influenzae* [35]. Moreover, antibiotic use is associated with increased prevalence of PNSP [12, 36–38] and BLNAR [39], but only some studies show antibiotic consumption to be a risk factor for carrying beta-lactamase producing *H.influenzae* [40–42] while others do not [31, 43, 44].

When we considered only the children with growth of either *S.pneumoniae* or *H.influenzae* (*n* = 178) we found that boys were more often colonised with resistant bacteria than girls, which is supported by some previous studies [36] but not by others [12]. Boys have also been shown to catch more RTIs in younger ages than girls [45, 46] and develop complications to AOM more often [47]. The reasons for these differences between the sexes are not known.

Other risk factors for carrying PNSP (MIC ≥ 0.125 mg/L) have been reported in previous studies with various results. Some studies support our findings that younger age predisposes to PNSP carriage [12, 31, 36], and also longer duration of PNSP carriage [36] Other studies show that attending a day care centre increases the risk [31] while the number of siblings, presence of respiratory tract disease and parental smoking did not affect resistance levels in the studies mentioned above, nor in our results.

A lower PNSP prevalence in a PHC population compared to local microbiology laboratory data, as was seen in Iceland in 1993 [11, 12] may be due to patient selection. In the Swedish guidelines, it is only recommended to perform NP swabs on patients with RTIs when first-line treatment has failed or in recurrent infections. The local microbiology laboratory data also includes cultures from hospital patients, both admitted and attending Emergency services and in some cases also patients referred from primary care. The differences in case-mix provide support for regular measurements of resistance in primary care populations to validate laboratory resistance data. In many counties in Sweden there has also been a relative reduction in the number of performed NP swabs (Swedres/ SWARM 2001-2016, www.folkhalsomyndigheten.se) and the cultured isolates therefore may exaggerate the prevalence of resistance, which in turn could have implications for treatment guidelines and empirical choice of antibiotics.

The carriage rates of *H.influenzae* were similar in our findings and the local microbiological laboratory data, as were the levels of beta-lactamase *producing H.influenzae* and BLNAR.

## Conclusion

The prevalence of resistant bacteria in the upper respiratory tract in young children in Swedish primary care was low. Phenoxymethylpenicillin can therefore still be recommended for empirical treatment. In order to provide a basis for empirical therapies resistance data from microbiological laboratories should be validated by regular studies on primary care populations. Our results support the fact that recent antibiotic treatment increases bacterial resistance in children and continuous work to reduce unnecessary antibiotic prescribing should be prioritised.

## Additional files

Additional file 1: Parental questionnaire in English. (DOCX 25 kb)

Additional file 2: Parental questionnaire in Swedish. (DOCX 26 kb)

## Abbreviations

AOM: Acute otitis media
BLNAR: Beta-lactamase negative ampicillin resistant
Hib: *Haemophilus Influenzae* type b
NP: Nasopharynx
Penicillin V: phenoxymethylpenicillin
PHC: Primary Health Care
PHCC: Primary Health Care Centre
PNSP: Penicillin non-susceptible pneumococci
RTI: Respiratory tract infection
