# Supplementary material for: Children with respiratory tract infections in Swedish primary care; prevalence of antibiotic resistance in common respiratory tract pathogens and relation to antibiotic consumption
Source: BMC Infect Dis. 2017 Sep 4;17:603. doi: 10.1186/s12879-017-2703-3 (PMC5583975; doi:10.1186/s12879-017-2703-3)
Supplement: Supplementary file 1 — Parental questionnaire in English. (DOCX 25 kb) [file 12879_2017_2703_MOESM1_ESM.docx]

**Research study: Prevalence of resistant bacteria in children, age 0-10 years. Questionnaire to be completed by accompanying adult:**

**Age of child (years)**:______ girl□ boy□

1. **Main activity of child during daytime:**

School□ Preschool□ At home□ Don´t know□

2. **Number of household members:**_____

Number of children 0-6 years of age, in your household:____

3. **Smokers in the family?** Yes□ No□ Don´t know□

4. **Does the child have any previous illness in the airways, such as asthma, allergy or other? If yes, which illness**:______________________ No□ Don´t know□
5. **Is the child vaccinated against pneumococci?** Yes□ No□ Don´t know: □

6. **Has the child been admitted to hospital in the last 6 months?**
Yes□ No□ Don´t know: □

7. **Has the child travelled abroad in the last 3 months?** Yes□ No□ Don´t know: □

8. **How many treatments with antibiotics has the child received during the last year?**
 0□ 1-2□ 3 or more□ Don´t know: □
9. **Has the child been treated with antibiotics the last 4 weeks?**
 Yes□ **Name of antibiotic:______________** No□ Don´t know: □

10. **Which symptoms does the child present with today?**
cough□ cold□ sore throat□ ear ache□ fever□
other□ Don´t know□

11. **Was the child prescribed antibiotics at today’s visit?**

**If yes, which antibiotic?______________________**  No□ Don´t know□

12. **Approval of researchers’ access to medical register?** Yes□ No□
13. **Choose to participate with swab from the nose**. Yes□ No□

**Thank you for your time!**

**Please, leave the questionnaire in the reception or lab.**
