# Supplementary material for: Children with respiratory tract infections in Swedish primary care; prevalence of antibiotic resistance in common respiratory tract pathogens and relation to antibiotic consumption
Source: BMC Infect Dis. 2017 Sep 4;17:603. doi: 10.1186/s12879-017-2703-3 (PMC5583975; doi:10.1186/s12879-017-2703-3)
Supplement: Supplementary file 2 — Parental questionnaire in Swedish. (DOCX 26 kb) [file 12879_2017_2703_MOESM2_ESM.docx]

**Forskningsstudie för kartläggning av motståndskraftiga bakterier hos barn, 0-10 år. Frågor till vårdnadshavare/medföljande vuxen:**

**Barnets ålder (hela år):_____** Flicka□ Pojke□

1. **Var vistas barnet dagtid?**

□Skola □Fritids □ Förskola □ Dagmamma □ Hemma enbart □Vet ej

1. **Hur många bor i ert hushåll?____**

Hur många är barn 0-18 år?____

1. **Röker någon i familjen?** Ja□ Nej□ Vet ej□
2. **Har barnet någon bakomliggande sjukdom i luftvägarna (tex astma, allergi eller annat)?**

Ja, vad:__________________________ Nej□ Vet ej□

1. **Är barnet pneumokockvaccinerat? (ingår i basprogrammet på BVC sedan 2009)**

Ja□ Nej□ Vet ej□

1. **Har barnet varit inlagd på sjukhus de senaste 6 månaderna?** Ja Nej□ Vet ej□
2. **Har barnet varit utomlands de senaste 3 månaderna?** Ja □ Vilket land?__________ Nej□ Vet ej□
3. **Hur många kurer med antibiotika har barnet använt det senaste året:**

0:____1-2:_____ >3:_____ Vet ej□

1. **Har barnet fått antibiotika de senaste 4 veckorna?**

Ja□ Vilket preparat?________________ Nej□ Vet ej□

1. **Vilka besvär från luftvägarna gör att ni söker läkare idag?**

Hosta□ Snuva□ Halsont□ Ont i öronen□ Feber□ Annat: _____ Vet ej□

1. **Förskrevs barnet antibiotika vid dagens läkarbesök?**

Om, ja, vilket preparat?_________________ Nej□ Vet ej□

1. **Medgivande att gå in i läkemedelsförteckningen (apotekets register för utskrivna läkemedel).**

Ja□ Nej□

1. **Lämnar prov från näsan (nasopharynxodling) idag:** Ja □ Nej□

Tack för besväret!

***Vänligen lämna lappen till den som tar näsprovet (nasopharynxodlingen.)***
